# Adjuvant Chemotherapy Improves Survival for Children With Massive Choroidal Invasion of Retinoblastoma

**DOI:** 10.1167/iovs.64.11.27

**Published:** 2023-08-21

**Authors:** Zhao Xun Feng, Junyang Zhao, Nan Zhang, Mei Jin, Brenda Gallie

**Affiliations:** 1 Department of Ophthalmology, University of Ottawa, Ottawa, Canada; 2 Pediatric Oncology Center, Beijing Children's Hospital, Capital Medical University, Beijing, China; 3 Department of Ophthalmology, Beijing Children's Hospital, Capital Medical University, Beijing, China; 4 Department of Pathology, Beijing Children's Hospital, Capital Medical University, Beijing, China; 5 Department of Medical Oncology, Beijing Children's Hospital, Capital Medical University, Beijing, China; 6 Beijing Key Laboratory of Pediatric Hematology Oncology, Key Laboratory of Major Diseases in Children, Beijing, China; 7 Department of Ophthalmology, Hospital for Sick Children, Toronto, Canada; 8 Krembil Research Institute and Techna Institute, University Health Network, Toronto, Canada; 9 Departments Ophthalmology, Medical Biophysics and Molecular Genetics, University of Toronto, Toronto, Canada

**Keywords:** retinoblastoma, massive choroidal invasion (MCI), chemotherapy, survival, relapse

## Abstract

**Purpose:**

The purpose of this study was to investigate the effect of adjuvant chemotherapy on outcomes of children with massive choroidal invasion (MCI).

**Methods:**

In this study, we **r**eviewed the 5-year relapse-free survival (RFS) and overall survival (OS) of children diagnosed with MCI, managed with or without adjuvant chemotherapy. Excluded were children with additional other high-risk features (post-laminar optic nerve invasion, scleral invasion, or overt extraocular disease).

**Results:**

Of 3566 children diagnosed with retinoblastoma, 2023 had enucleation, and 60 eyes of 60 children had pathology showing MCI without concomitant high-risk features. Enucleation was primary (22, 37%), or secondary (38, 63%) after failed eye salvage. Adjuvant systemic chemotherapy (median = 4, range = 1–8 cycles) was given to 48 of 60 (80%) children; 12 of 60 (20%) children had no adjuvant therapy. Five-year RFS was 88.5% (95% confidence interval [CI] = 79.7%–97.3%) and 5-year OS was 90.1% (95% CI = 81.7%–98.5%). Pre-enucleation chemotherapy did not affect RFS (89.7% vs. 75.0%; *P* = 0.657). Adjuvant chemotherapy improved RFS (97.2% vs. 55.6%; *P* < 0.001) and OS (97.2% vs. 66.7%; *P* < 0.001). In subgroup analysis, adjuvant chemotherapy improved RFS for both primarily enucleated (5-year RFS 100% vs. 50.0%; *P* = 0.002) and secondarily enucleated children (5-year RFS 95.8% vs. 60.0%; *P* = 0.005). The number of children treated with adjuvant chemotherapy to prevent one post-enucleation systemic relapse or death is three.

**Conclusions:**

Adjuvant chemotherapy significantly decreased the risk of tumor relapse and death for children with pathological MCI. For every three children treated with adjuvant chemotherapy, one systemic relapse or death could be prevented.

Retinoblastoma is a primary intraocular malignancy and the most common ocular cancer affecting children worldwide.^1^^,^^2^ Although there have been significant advances in eye salvage treatment modalities,^3^^–^^6^ enucleation remains a crucial treatment option for eyes with high-risk clinical features, no vision potential, or failed salvage therapies. Following enucleation, histopathology can identify eyes at high-risk of extraocular extension which guide adjuvant treatment.^7^ The goal of adjuvant chemotherapy is to treat microscopic tumor metastasis prior to clinically apparent disease to improve patient survival. One of such high-risk features that may warrant treatment is massive choroidal invasion (MCI), which is defined by the International Retinoblastoma Staging Working Group as a maximum diameter of invasive tumor focus of 3 mm or more, or full-thickness contacting sclera.^8^ Systemic chemotherapy is known to induce bone marrow suppression, autotoxicity, and nephrotoxicity.^9^^,^^10^ The decision to pursue adjuvant chemotherapy requires careful balance between risk of extraocular relapse and chemotherapy-related complications. The management of children with MCI remains controversial, and, to date, no study has provided statistical evidence of survival benefit with adjuvant chemotherapy for MCI. We summarized published case series reporting survival of children with massive choroidal invasion in Table 1.^11^^–^^27^

**Table 1.** Published Series Reporting Survival of Children With Massive Choroidal Invasion

Authors	Publication	Adjuvant Chemotherapy	Primary Treatment	Number of Children	Recurrence-Free Survival	Overall Survival	Follow-Up (Mo)	Comments
Feng et al.	(Present)	No	Enucleation, IVC or IAC	48	98%	98%	62.1 (median)	
Feng et al.	(Present)	Yes	Enucleation, IVC or IAC	12	58%	67%		
Kashyap et al.	Asia Pac J Clin Oncol. 2021 April;17(2):e100-e108	NA	Enucleation	157	NA	95%	NA	Included children with other concomitant high-risk features
Kaliki et al.	Eye (Lond). 2020 Aug;34(8):1441-1448	95% of high-risk RB received chemo	Enucleation	120	103/120 (86%)	103/120 (86%)	36 (median)	Included children with other concomitant high-risk features
Kaliki et al.	Retina. 2018 Oct;38(10):2023-2029	Most received chemo (>95%)	Enucleation	69	64/69 (93%)	65/69 (94%)	Indian: 28 (median) American: 49 (median)	Included children with other concomitant high-risk features
Kaliki et al.	Ophthalmology. 2015 Jun;122(6):1165-72	96% of high-risk RB received chemo	Enucleation	69	66/69 (96%)	66/69 (96%)	28 (median)	Included children with other concomitant high-risk features
Bosaleh et al.	Arch Ophthalmol. 2012 Jun;130(6):724-9	No	Enucleation or IVC	31	29/31 (93%)	30/31 (96%)	44 (median)	Isolated massive choroidal invasion
Bosaleh et al.	Arch Ophthalmol. 2012 Jun;130(6):724-9	Yes	Enucleation	4	4/4 (100%)	4/4 (100%)		
Chantada et al.	JAMA Ophthalmol. 2013 Sep;131(9):1127-34	No	NA	33	94%	NA	78 (median)	Isolated massive choroidal invasion
Lu et al.	Br J Ophthal. 2019 Sept;103(9)1272-1277	Mixed	Enucleation	31	27/31 (87%)	28/31 (90%)	60.0 (mean)	Included children with other concomitant high-risk features
Singh et al.	Int J Clin Oncol. 2016 Aug;21(4):651-657	NA	Enucleation	31	NA	84%	NA	Included children with other concomitant high-risk features
Singh et al.	Clin Exp Ophthalmol. 2015 Aug;43(6):550-7	NA	Enucleation	20	NA	80%	NA	Included children with other concomitant high-risk features
Suryawanshi et al.	Arch Pathol Lab Med. 2011 Aug;135(8)1017-23	Mixed	IVC	17	10/17 (59%)	NA	24 (mean)	Included children with other concomitant high-risk features
Loya et al.	J AAPOS. 2023 Feb;27(1):32.e1-32.e8	NA	NA	10	NA	100%	72.2 (mean)	Included children with massive choroidal invasion without PLONI
Chantada et al.	J Pediatr Hematol Oncol. 2009 May;31(5):325-9	No	Enucleation	10	9/10 (90%)	NA	51 (median)	Isolated massive choroidal invasion
Yousef et al.	Turk Patoloji Derg. 2014;30(3):171-7	Mixed	Enucleation	9	9/9 (100%)	9/9 (100%	40 (median)	Included children with other concomitant high-risk features
Chévez-Barrios et al.	J Clin Oncol. 2019 Nov 1;37(31):2883-2891	Yes	Enucleation	6	5/6 (83%)	100%	48.0 (median)	Isolated massive choroidal invasion
Nawaiseh et al.	Turk Patoloji Derg. 2015;31(1):45-50	Yes	Enucleation	6	6/6 (100%)	6/6 (100%)	36 (median)	Included children with other concomitant high-risk features
Fabian et al.	Ophthalmology. 2017 Jun;124(6):851-858	Yes	Enucleation or IVC	5	5/5 (100%)	5/5 (100%)	73.2 (median)	One child has concomitant superficial scleral invasion
Laurent et al.	JAMA Ophthalmol. 2016 Dec 1;134(12):1374-1379	No	Enucleation, IVC or IAC	5	5/5 (100%)	5/5 (100%)	NA	Isolated massive choroidal invasion

Included series were published 2003 or later (concurrent with our study population) with minimum cohort size of 5 eyes.

NA, not applicable; PLONI, post-laminar optic nerve invasion; RB, retinoblastoma.

To address this gap, we conducted a retrospective study to evaluate the effect of adjuvant chemotherapy on relapse-free survival (RFS) and overall survival (OS) in children with isolated MCI. The primary objective of the study was to determine whether adjuvant chemotherapy had a significant impact on the survival outcomes of these children. Secondary objectives included assessing the effects of pre-enucleation chemotherapy and concomitant low-risk histopathological features on survival. Our findings provide important insights into the optimal management of children with MCI to prevent extraocular relapse and improve survival rates.

## Patients and Methods

### Study Design

**Figure 1.**
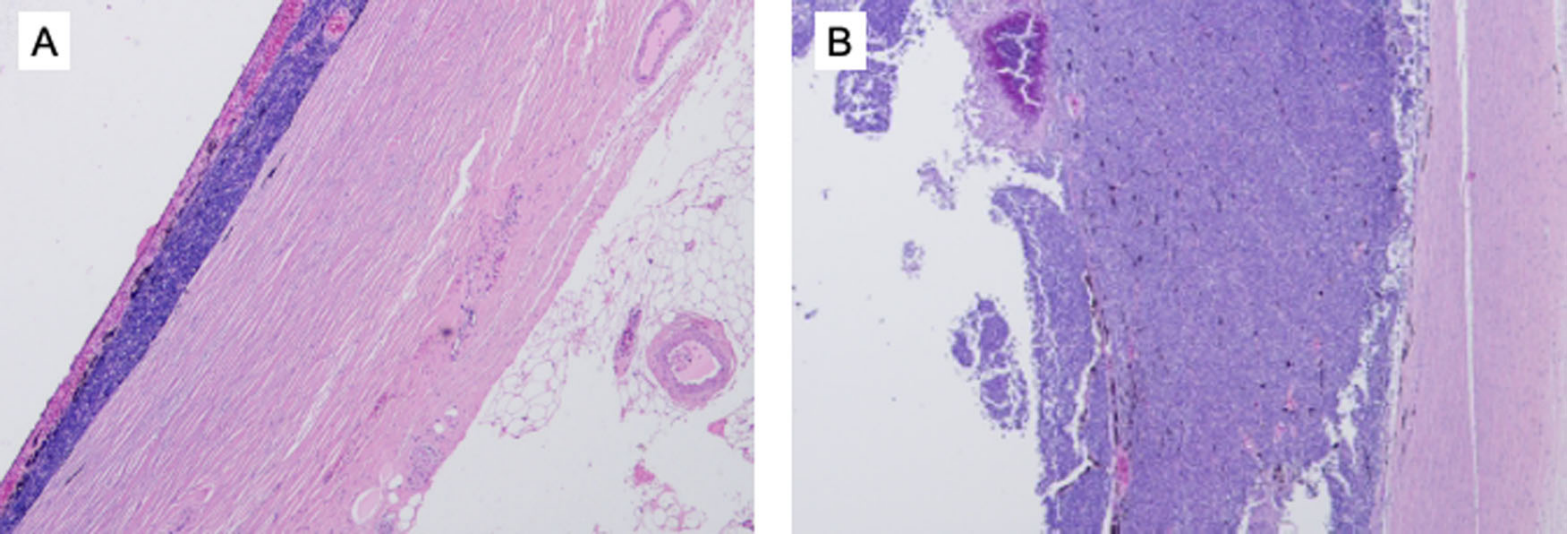
Massive choroidal invasion. (**A**) choroidal invasion >3 mm. (**B**) full thickness choroidal invasion.

This is a retrospective cohort study approved by the Institutional Ethics Committee at Beijing Children's Hospital in accordance with the Declaration of Helsinki. We reviewed records of all children managed by our team at 29 Chinese treatment centers between January 2007 and January 2019. Inclusion criteria was MCI on histopathology, defined as choroid invasion >3 mm in largest diameter, or any full-thickness choroidal involvement (Fig. 1). Children with additional pathologic risk factors (post-laminar optic nerve invasion, scleral invasion, or overt extraocular disease) were excluded.

All patients were managed by one retinoblastoma team led by author Junyang Zhao (a retinoblastoma specialist), who participates in care with local ophthalmologists at 29 Chinese centers. Clinical data collected included sex, laterality, first sign, International Intraocular Retinoblastoma Classification (IIRC), American Joint Committee on Cancer (AJCC) classification, treatments before and after enucleation, location of metastasis and dates of diagnosis, last follow-up, metastasis, and death. Last follow-up date for this study was December 1, 2021. Lost to follow-up was defined as missing two consecutive appointments and unable to contact the parent. Prior to enucleation, all children had routine magnetic resonance imaging (MRI) or computed tomography, when MRI is not available, to screen for extraocular disease.

### Pathology Review

Pathology slide preparation was performed either at affiliated academic centers or the patient's local hospitals. For enucleation at affiliated centers, representative microscopic sections and/or gross images were sent to and reviewed by ocular pathologist at Beijing Children's Hospital. For enucleation at patient's local hospital, only pathology reports were available for review. Treatment decisions were based on the pathology report at Beijing Children's Hospital whenever available. Choroidal invasion was classified as focal (<3 mm, pT1) or massive (>3 mm or full-thickness, pT3a, and MCI). Optic nerve invasion was classified as pre-/intra-laminar; post-laminar, but not to transected margin, or tumor at the transected end. Anterior segment invasion, scleral invasion, and extraocular invasion were also identified.

### Chemotherapy Regimen

The indication of pre-enucleation chemotherapy for the majority of children was attempted eye salvage. Enucleation was performed in these children after unsuccessful eye salvage due to either residual active tumor or recurrent tumor. Some patients received neoadjuvant chemotherapy, defined as a short course of chemotherapy (systemic, intra-arterial, and/or intravitreal) followed by planned enucleation. Neoadjuvant chemotherapies were offered to children whose parents initially refused enucleation or eyes with significant risk of surgical bleeding due to high tumor burden (vitreous hemorrhage, iris neovascularization, hyphema, and hemorrhagic chemosis). Decisions for adjuvant chemotherapy were at the discretion of the retinoblastoma specialist, medical oncologist, and the children's parents. The systemic chemotherapy regimen was intravenous carboplatin 560 mg/m^2^ on day 1, etoposide 150 mg/m^2^ on days 1 and 2, or teniposide 230 mg/m^2^ on day 2, and vincristine 1.5 mg/m^2^ on day 2, on a 28 day/cycle. There was variation in the number of chemotherapy cycles due to a multitude of factors, including treatment of the contralateral eye, poor response to chemotherapy, short course of neoadjuvant chemotherapy, parental choice (financial constraint, concern about side effects, or perception that the disease was cured), institutional protocols, and medical contraindications to chemotherapy (severe thrombocytopenia, neutropenia, or adverse event from chemotherapy).

### Statistical Analysis

Continuous and categorical variables were compared using Mann-Whitney *U* test and chi-squared test, respectively. Kaplan-Meier was used to estimate RFS and OS, with the log-rank test to compare survival between groups. RFS is defined as the length of time after enucleation that the patient survives without disease recurrence. All reported *P* values are 2-sided, and < 0.05 indicated significance. All analyses were performed using SPSS Version 25 (IBM Corp, Armonk, NY, USA).

## Results

### Demographic and Clinical Characteristics

Of 3566 children with retinoblastoma managed by our team across 29 treatment centers during the study period, 2023 had enucleation. MCI without other high-risk histopathology features was present in 60 eyes of 60 children (all data in the Supplementary Table). Affiliated academic centers performed 54 of 60 enucleations and pathology slides preparation; 6 were performed at the patient's local hospital. Of 60 children with MCI, adjuvant chemotherapy was given to 48 of 60 (80%) children (median = 4 cycles, range = 1–8); 15 of 48 (31%) children received 3 cycles or less. Clinical characteristics of children with and without adjuvant chemotherapy are summarized in Table 2. Median follow-up was 62.1 months (range = 4.1–138.9) since diagnosis. Five children (8%) were lost to follow-up; none had an active tumor at the last follow-up.

**Table 2.** Baseline Clinical Characteristics of Studied Children and Eyes With and Without Adjuvant Chemotherapy

Characteristic	With Adjuvant Chemotherapy (*n* = 48)	Without Adjuvant Chemotherapy (*n* = 12)	*P* Value
Sex			
Male	26 (54%)	7 (58%)	0.749
Female	22 (46%)	5 (42%)	
Age of diagnosis (mo)			
Median	20	20	0.897
Range	2–76	5–44	
Laterality			
Unilateral	43 (90%)	10 (83%)	0.546
Bilateral	5 (10%)	2 (17%)	
Enucleation			
Primary	20 (42%)	2 (17%)	0.108
Secondary	28 (58%)	10 (83%)	
IIRC of studied eye			
D	17 (35%)	10 (83%)	0.012*
E	30 (63%)	2 (17%)	
Unknown	1 (2%)	0	
AJCC cTNM of studied eye			
cT2b	17 (35%)	10 (84%)	0.054
cT3b	8 (17%)	1 (8%)	
cT3c	9 (19%)	0	
cT3d	13 (27%)	1 (8%)	
Unknown	1 (2%)	0	

IIRC = International Intraocular Retinoblastoma Classification; AJCC = American Joint Committee on Cancer.

### Pre-Enucleation Therapy

Enucleation was primary in 22 (37%) children and secondary in 38 (63%). Pre-enucleation therapies were systemic chemotherapy (34; median = 5 cycles, range = 1–11), intra-arterial chemotherapy (15; median = 2 cycles, range = 1–5), intravitreal chemotherapy (7; median = 2 cycles; range = 1–6), tylectomy (12), and external beam radiation (2). Many patients received a combination of these therapies prior to secondary enucleation. Median time from diagnosis to enucleation was 4.9 months (range = 0–43.6), significantly longer for children with secondary enucleation than those primarily enucleated (10.8 vs. 0 months, *P <* 0.001).

### Histopathology

Concomitant low-risk features were present in 32 of 60 (53%) eyes with MCI: pre/intra-laminar optic nerve invasion (26), anterior chamber invasion (12), or both features (6). Given the exclusion criteria, none had a high-risk feature other than MCI.

### Post-Enucleation Relapse and Mortality

Post-enucleation relapse in six (10%) children involved the orbit (1), spine (1), skull (1), abdomen (1), cerebral spinal fluid (1), and brain (1; Table 3). Of the children who relapsed, 5 of 6 died. One child with orbital relapse survived after exenteration, external beam radiation, intrathecal chemotherapy, and high-dose systemic chemotherapy. Of five children who died, one had bilateral disease and the non-enucleated eye had no active tumor at last follow-up; therefore, all deaths were attributed to tumor relapse from the enucleated eye. Children with or without relapse were not significantly different in sex (girls, 67% vs. 43%; *P* = 0.261), age at diagnosis (21 vs. 20 months; *P* = 0.646), laterality (unilateral, 89% vs. 83%; *P* = 0.688), and rate of primary enucleation (17% vs. 39%; *P* = 0.284). There is a lower proportion of group E eyes among children with tumor relapse than those without; however, this did not meet statistical significance (17% vs. 57%; *P* = 0.058). Of six children who developed relapse, the pre-enucleation treatments were systemic chemotherapy (5/6), intra-arterial chemotherapy (1/6), intravitreal chemotherapy (1/6), and tylectomy (2/6). Median time from enucleation to relapse was 5.8 months (range = 1.5–8.1 months), and to metastatic death was 10.9 months (range = 2.6–21.1 months).

**Table 3.** Summary of Patients With Relapse

Patient	Pre/Intra-Laminar ON Invasion	AS Invasion	Adjuvant Chemotherapy	Location of Relapse	Time From Enucleation to Relapse (Mo)	Outcome	Time From Enucleation to Death (Mo)
1	No	No	No	Orbit	3.0	Alive	N/A
2	Yes	Yes	No	Spine	3.7	Died	10.9
3	No	Yes	Yes	Skull	8.0	Died	21.1
4	Yes	No	No	Abdomen	8.1	Died	12.1
5	Yes	No	No	CSF	1.5	Died	2.6
6	No	No	No	Brain	5.8	Died	7.5

ON = optic nerve; AS = anterior segment; CSF = cerebrospinal fluid.

**Figure 2.**
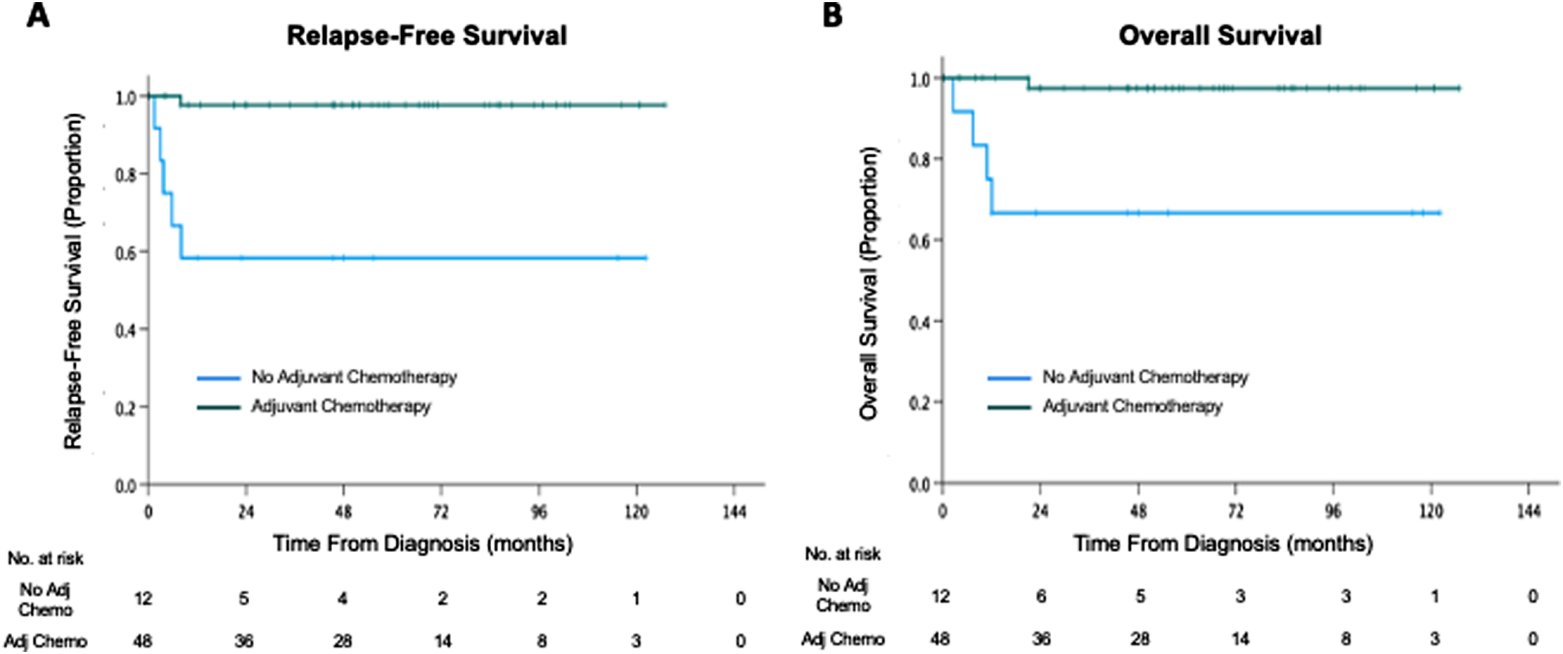
Kaplan-Meier curves of (**A**) relapse-free survival and (**B**) overall survival of children with and without adjuvant chemotherapy.

The 5-year RFS was 89.2% (95% confidence interval [CI] = 81.0%–97.4%) and 5-year OS was 90.1% (95% CI = 82.8%–98.4%; Fig. 2). Children who received adjuvant chemotherapy had significantly higher RFS than those who did not (5-year RFS 97.7% vs. 58.3%; *P* < 0.001). In subgroup analysis, RFS was higher for children who received adjuvant chemotherapy than those who did not for both primarily enucleated (5-year RFS 100% vs. 50.0%; *P* = 0.002) or secondarily enucleated children (5-year RFS 95.8% vs. 60.0%; *P* = 0.005). The OS of children who received adjuvant chemotherapy was significantly higher than of those who did not (5-year OS 97.4% vs. 66.7%; *P* < 0.001). The number needed to treat to prevent one post-enucleation relapse or death is three.

RFS was not significantly different between children with unilateral versus bilateral retinoblastoma (5-year RFS 90.1% vs. 80.0%; *P* = 0.466), or primary enucleation versus secondary enucleation (5-year RFS 95.5% vs. 85.4%; *P* = 0.267). Concomitant pre-/intra-laminar invasion (5-year RFS 88.5% vs. 89.9%; *P* = 0.856) or anterior chamber invasion (5-year RFS 78.8% vs. 91.3%; *P* = 0.266) did not significantly affect RFS.

## Discussion

The management of children with MCI in retinoblastoma is currently debated, as the potential benefit of adjuvant chemotherapy must be weighed against the risk of complications associated with systemic chemotherapy.^9^^,^^10^^,^^28^^–^^32^ Death from systemic chemotherapy complications is not uncommon in children with retinoblastoma.^33^^–^^36^ Our study, based on a cohort of 60 children with MCI, provides important evidence on the survival benefit of adjuvant chemotherapy for this high-risk feature. We observed that children who received adjuvant systemic chemotherapy had significantly higher RFS and OS than those who did not. For every three children treated with adjuvant chemotherapy, one extraocular relapse or death would be prevented.

Our findings are consistent with guidelines from several countries, which recommend adjuvant chemotherapy for MCI.^37^^,^^38^ Guidelines from Austria, Germany, Czech Republic, Israel, the Netherlands, Spain, and the United Kingdom recommend between three and six cycles of vincristine and cyclophosphamide for MCI. Guidelines from France and Switzerland recommend two cycles of vincristine and cyclophosphamide for MCI. Nevertheless, other centers suggest that adjuvant chemotherapy can be withheld for children with isolated choroidal invasion based on the low rate of extraocular relapse and the potential for post-relapse rescue.^39^

Our study supports prophylactic adjuvant chemotherapy for children with MCI, as extraocular relapse and intensive rescue therapy are associated with significantly increased mortality, which likely outweighs the harm of unnecessary chemotherapy. In our study, more group E children received adjuvant chemotherapy than group D children likely owing to their parent's acceptance of chemotherapy because the disease was more advanced. Although higher IIRC staging generally confers worse survival, because a higher proportion of group E children received adjuvant treatment, they had fewer systemic relapses than group D children.

In a Children's Oncology Group multicenter prospective trial, concomitant peripapillary MCI and 1.5 mm or greater of post-laminar optic nerve invasion had the poorest outcome: 3 of 15 (20%) children developed recurrence.^24^ Because our present study excluded children with post-laminar optic nerve invasion, we cannot corroborate this finding. We found the concomitant presence of low-risk features (pre-/intra-laminar optic nerve invasion and anterior chamber invasion) did not significantly affect RFS.

Our prior publication showed pre-enucleation chemotherapy interferes with assessment of histopathology.^33^^,^^40^ The danger is that pre-enucleation chemotherapy may down-stage high-risk features to low-risk, reducing surveillance and adjuvant management, and increasing risk of metastatic death. In our present cohort of children with apparent isolated high-risk MCI, pre-enucleation chemotherapy may have masked other high-risk features (e.g. scleral invasion). Nevertheless, our data showed adjuvant chemotherapy decreases relapse for children following both primary (5-year RFS 100% vs. 50.0%; *P* = 0.002) or secondary enucleation (5-year RFS 95.8% vs. 60.0%; *P* = 0.005).

A prospective study by the Children's Oncology Group demonstrated considerable discrepancy between pathology review by contributing center and central consensus pathology review.^24^ Our study reported on pathology staging performed in routine clinical care in China and is limited by the lack of consensus pathology review. We attempt to have all pathology slides reviewed by one ocular pathology department in Beijing whenever possible; however, slides from peripheral hospitals are not always available due to real-world limitations. Furthermore, our study is also limited by its retrospective study design and non-uniform chemotherapy cycles. Although we could not statistically assess the optimal number of treatment cycle given sample size limitation, we suggest four to six cycles are a reasonable choice for children with MCI to avoid extraocular relapse. Those with MCI following secondary enucleation may require closer monitoring for relapse due to higher risk of chemotherapy-induced drug resistance and masking of higher-risk pathology features. Future studies could investigate the least aggressive regimen for MCI that does not compromise survival and the effectiveness of novel targeted therapies that may avoid the systemic toxicity associated with traditional chemotherapy.

## Conclusions

In conclusion, our study provides important evidence supporting the use of adjuvant chemotherapy for children with isolated MCI of retinoblastoma. We found that children who received adjuvant chemotherapy had significantly higher rates of RFS and OS than those who did not. Furthermore, our finding suggests that for every three children treated with adjuvant chemotherapy, one relapse or death could be prevented. Our results have significant clinical implications for the management of children with MCI and underscore the importance of balancing the risk of extraocular relapse against the potential toxicities of chemotherapy. We recommend clinicians discuss the benefits and risks of adjuvant chemotherapy with parents and provide appropriate counseling to those who refuse the treatment.

## Supplementary Material

Supplement 1
